# Engineered apoptotic vesicle mimetics with tunable “eat-me” signaling precisely regulate tumor-associated macrophages for potentiating cancer immunotherapy

**DOI:** 10.1016/j.apsb.2025.11.032

**Published:** 2025-11-29

**Authors:** Yu Liu, Chunbai Xiang, Yeneng Dai, Chao Li, Michael N. Okeke, Ting Jiang, Xing Yang, Yehuda G. Assaraf, Kai Miao, Yue Wang, Zhiwei Zhang, Duo Zhang, Yaping Li, Ping Gong, Qi Zhao

**Affiliations:** a MoE Frontiers Science Center for Precision Oncology, Cancer Center, Faculty of Health Sciences, University of Macau, Taipa, Macau 999078, China; b Key Laboratory of Biomedical Imaging Science and System, Chinese Academy of Sciences, State Key Laboratory of Biomedical Imaging Science and System, Guangdong Key Laboratory of Nanomedicine, CAS–HK Joint Lab for Biomaterials, Shenzhen Institutes of Advanced Technology, Shenzhen 518055, China; c University of Chinese Academy of Sciences, Beijing 100049, China; d State Key Laboratory of Drug Research & Center of Pharmaceutics, Shanghai Institute of Materia Medica, Chinese Academy of Sciences, Shanghai 201203, China; e Faculty of Pharmaceutical Sciences, Shenzhen University of Advanced Technology, Shenzhen 518107, China; f The Fred Wyszkowski Cancer Research Laboratory, Faculty of Biology, Technion-Israel Institute of Technology, Haifa 3200003, Israel

**Keywords:** Tumor-associated macrophages, Apoptotic vesicle, pH-responsive PEG coating, cGAS–STING pathway, SIRP*α*, Repolarization, Phagocytosis, Immunotherapy

## Abstract

Repolarizing immunosuppressive M2-phenotype tumor-associated macrophages (TAMs) and blocking the CD47/SIRP*α* axis are promising strategies to enhance cancer immunotherapy. However, non-selective disruption of macrophage phenotypic balance and CD47/SIRP*α* signaling can lead to immune-related side effects. To address this, we develop a smart biomimetic nanoparticle (PARM) loaded with R848 and manganese ions (Mn^2+^). PARM is coated with an apoptotic vesicle membrane and a pH-sensitive PEG corona, enabling targeted delivery to TAMs in the acidic tumor microenvironment (TME). The PEG corona protects the nanoparticle from uptake during circulation and sheds in the TME, exposing the apoptotic vesicle membrane. This triggers specific recognition and uptake by TAMs *via* the “eat-me” signal. R848 and Mn^2+^ repolarize TAMs into a pro-inflammatory phenotype, while the activation of cGAS–STING pathway by Mn^2+^ reduces SIRP*α* expression and enhances TAM phagocytosis. *In vivo* studies demonstrate that PARM remodels the immunosuppressive TME by repolarizing TAMs and promoting CD8^+^ T cell infiltration. This leads to significant inhibition of tumor growth and metastasis. These findings highlight the multifaceted role of the cGAS–STING pathway in TAM modulation and present a novel strategy for enhancing macrophage-based cancer immunotherapy.

## Graphical abstract

This study constructed a pH-responsive sheddable PEG-shielded apoptotic vesicle-biomimetic R848/Mn^2+^ coordination nanoparticle termed PARM. PARM significantly inhibited tumor growth and metastasis by remodeling the immunosuppressed tumor microenvironment.

**Figure:**
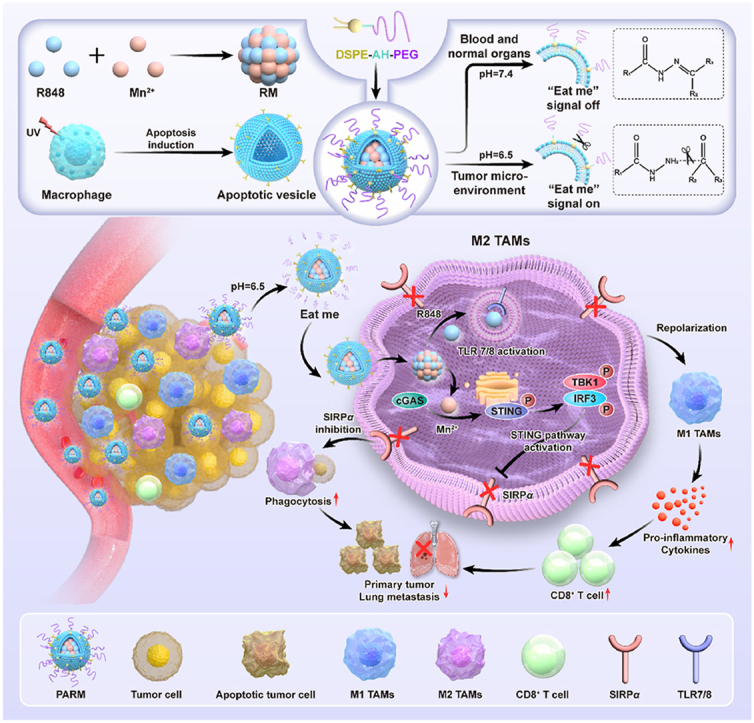

## 1 Introduction

Immunotherapy is an innovative and promising cancer treatment approach^1^, ^2^, ^3^, ^4^, ^5^. However, its clinical efficacy is still constrained by the immunosuppressive tumor microenvironment (TME)^6^^,^^7^. Tumor-associated macrophages (TAMs) constitute the major immune cell subset in the TME, predominantly exhibit an immunosuppressive M2-phenotype that drives immunosuppression and restricts immunotherapy efficacy^8^, ^9^, ^10^. Reprogramming protumoral M2-phenotype TAMs to antitumoral M1-phenotype has been shown to be effective for cancer immunotherapy^11^, ^12^, ^13^. Unfortunately, insufficient phagocytosis of tumor cells by TAMs continues to limit immunotherapy outcomes. Elevated expression of CD47 on tumor cells facilitates its recognition by signal-regulatory protein *α* (SIRP*α*) expressed on the surface of macrophages, leading to inhibition of phagocytosis^14^, ^15^, ^16^. Therefore, an ideal strategy to maximize the efficacy of macrophage-based immunotherapy requires synergistically regulating polarization and phagocytosis^17^, ^18^, ^19^.

To date, several drug delivery strategies that simultaneously enhance the M1-polarization and phagocytic ability of macrophages have been developed^20^, ^21^, ^22^. For typical examples, Zhang et al.^23^ constructed mesoporous silica nanoparticles for the co-delivery of Toll-like receptor 7/8 (TLR7/8) agonist R848 and SIRP*α* antibody to enhance macrophage-mediated cancer immunotherapy. Lin and colleagues developed a nanoscale metal-organic framework co-loaded with the TLR-7 agonist imiquimod and CD47 antibody to boost macrophage immune regulation and the activation of innate immunity^24^. Despite these advances, concerns regarding safety and effectiveness persist. Given the widespread distribution of macrophages and the importance of maintaining their phenotypic balance for systemic homeostasis, it is crucial and necessary to precisely regulate the polarization and phagocytosis of TAMs^25^^,^^26^. Furthermore, current strategies predominantly rely on macromolecular drugs (*e*.*g*., antibodies/peptides) to modulate the CD47/SIRP*α* axis extracellularly. This creates significant challenges when combining them with intracellular-acting agents needed for TAM reprogramming^23^^,^^27^. Therefore, modulating intracellular signaling pathways in macrophages to regulate SIRP*α* expression represents a more efficient therapeutic strategy.

The cyclic GMP–AMP synthase (cGAS)-stimulator of interferon gene (STING) signaling pathway plays a central role in innate immune response, and its activation has been shown to promote dendritic cell maturation and M2-macrophage repolarization, which is crucial in tumor immunotherapy^28^, ^29^, ^30^, ^31^, ^32^, ^33^, ^34^. What is even more exciting, recent studies have revealed that activation of the cGAS–STING pathway may downregulate SIRP*α* expression in macrophages, thereby enhancing their phagocytic ability toward tumor cells^35^. Several STING agonists have demonstrated promising antitumor efficacy in preclinical studies, among which manganese ions (Mn^2+^), an essential nutrient metal ion, have garnered significant attention due to their wide source, low price, and robust anti-tumor immune response^36^, ^37^, ^38^.

**Figure 1:**
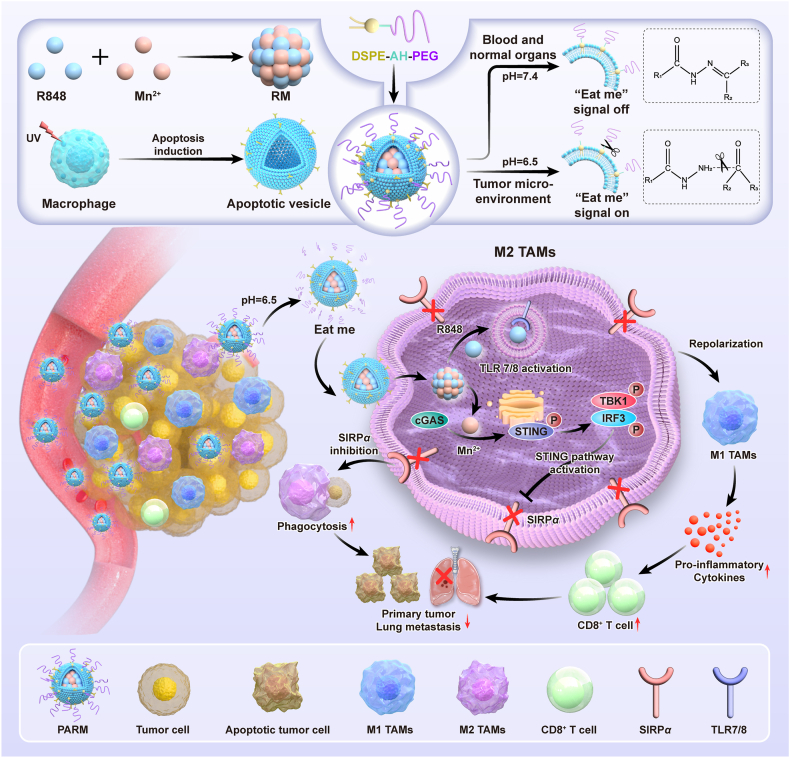
Schematic illustration of the preparation of engineered apoptotic vesicle mimetics (PARM) and their mechanism in precisely modulating the repolarization and phagocytosis of TAMs to potentiate cancer immunotherapy.

In this study, we developed an engineered apoptotic vesicle biomimetic nanoparticle termed PARM, which can precisely enhance M1-polarization and phagocytosis of TAMs. As illustrated in Fig. 1, the R848 and Mn^2+^ coordination nanoparticle was prepared as the core modified with apoptotic vesicle membrane on the surface, and further coated with DSPE-AH-PEG containing pH-sensitive acylhydrazone bond on the outermost layer. Due to the shielding effect of PEG, the nanoparticle remained stable in the neutral pH peripheral blood and normal organs. When the nanoparticle reached the acidic TME, the pH-sensitive bond was broken, thereby shedding the PEG corona, exposing the “eat me” signal of apoptotic vesicle, and was then taken up by TAMs^39^, ^40^, ^41^. R848 repolarized M2-phenotype TAMs into M1-phenotype. Mn^2+^ not only activated the cGAS–STING pathway to promote TAM repolarization, but also reduced the expression of SIRP*α*, thus enhancing the phagocytic activity of TAMs against tumor cells. PARM significantly reversed the immunosuppressive TME and displayed an excellent inhibitory activity on both primary and metastatic tumors. These findings support the role of the cGAS–STING pathway in modulating TAMs function and suggest a promising strategy for enhancing macrophage-based immunotherapy.

## 2 Materials and methods

### 2.1 Materials

Resiquimod (R848, 98%) and manganese chloride were purchased from bidepharm (Shanghai, China), H-151 was purchased from macklin (Shanghai, China), Indocyanine Green (ICG) was bought from InnoChem (USA) and DSPE-AH-PEG was obtained from Ruixi Biological Technology Co., Ltd. (Xi'an, China). Cytokine IL-4, IFN-*α* and IFN-*β* were bought from PeproTech (USA). PE-anti F4/80 (AB_3082990), FITC-anti CD206 (AB_10900988), APC-anti CD86 (AB_3106041), PerCP-anti CD8 (AB_893423), FITC-anti CD3 (AB_312661) were obtained from BioLegend (USA). Anti SIRP*α* (AB_3676758), TBK-1 (AB_776632), IRF-3 (AB_11155653), STING (AB_3086730) and GAPDH (AB_2107448) primary antibody and horseradish peroxidase (HRP)-conjugated secondary antibody (AB_2819160) were obtained from abcam (UK). Anti-phosphorylated TBK1 (AB_10693472), phosphorylated IRF3 (AB_823547) and phosphorylated STING (AB_2799831) primary antibody were purchased from CST (USA). Annexin V-FITC, Hoechst 33342, DAPI, RIPA lysates, Cell Counting Kit-8 (CCK-8) and BCA Rapid Protein Assay Kit were bought from Beyotime (Shanghai, China). FastPure Cell/Tissue Total RNA Isolation Kit was obtained from Vazyme Biotech Co., Ltd. (Nanjing, China). IL-6, TNF-*α*, IFN-*γ* and IFN-*β* ELISA Kits were purchased from MLBio (Shanghai, China). Nitric oxide (NO) content detection kit was obtained from Nanjing Jiancheng Bioengineering Research Institute (Nanjing, China). The cell culture components were purchased from Gibco (USA).

### 2.2 Cell culture

Raw 264.7 cells (CSTR: 19375.09.3101MOUSCSP5036) and 4T1 cells (CSTR:19375.09.3101MOUSCSP5056) were both purchased from the Cell Bank of the Committee on Type Culture Collection, Chinese Academy of Sciences (Shanghai, China). Wild-type Raw 264.7 cells and STING-knockout Raw 264.7 cells were cultured in Dulbecco's modified Eagle's medium (DMEM). 4T1 cells and GFP labeled 4T1 cells were cultured in RPMI-1640 medium. In addition, 10% fetal bovine serum (FBS) and 1% penicillin/streptomycin (P/S) were added to all medium. The cell culture conditions were set at 37 °C and 5% CO_2_.

### 2.3 Animals

Animal research was conducted in compliance with the Animal Care and Use Committee of Shenzhen Institutes of Advanced Technology, Chinese Academy of Sciences, and performed in accordance with the approved protocol (SIAT-IACUC-20230712-YYS-NMZX-CLT-1-02). Female BALB/c mice aged 6–8 weeks were purchased from Guangdong Yaokang Biotechnology Co., Ltd. and used for all animal experiment.

### 2.4 RNA-sequencing

To test the RNA-sequencing of macrophages with Mn^2+^ treatment, Raw 264.7 cells (2 × 10^5^/well) were incubated with MnCl_2_ (Mn^2+^, 2 μg/mL) for 12 h, the cells were then collected and treated with Trizol for subsequent RNA sequencing.

### 2.5 Analysis of the effects of Mn^2+^ on SIRPα expression

The Raw 264.7 cells (2 × 10^5^/well) were cultured in six-well plates and treated with MnCl_2_ (Mn^2+^, 0.5, 2 and 5 μg/mL) for 12 h. Then the cells were collected and protein samples were extracted *via* RIPA lysates. After the protein samples undergo electrophoresis, transfer, blocking, antibody incubation, and washing, the PVDF film was exposed and developed using the GelView 6000Pro II Multi-functional image workstation. The Western blot assay procedures for STING and SIRP*α* proteins in wild-type Raw 264.7 cells and STING-knockout Raw 264.7 cells were roughly the same as those described above.

For the mRNA expression levels detected by qPCR assay, total RNA was extracted from cells after different treatments, and cDNA was obtained by reverse transcription. cDNA was mixed with primers and other necessary qPCR reagents, and then quantitative RT-PCR was performed. The primer sequence is as follows:

SIRP*α*: F: 5′-CGCCAGGGAAATAACCCAGA-3′;

R: 5′-GTGGACCATGTCCAGGTCAG-3′;

*β*-Actin: F: 5′-CCACACCCGCCACCAGTTCG-3′;

R: 5′-TACAGCCCGGGGAGCATCGT-3′

### 2.6 Preparation and characterization of PARM

For the preparation of PEG-stabilized R848-manganese coordination nanoparticles (PRM), the MnCl_2_ aqueous solution (Mn^2+^ 10 mmol/L, 3 mL) was added drop by drop to R848 (2 mg/mL, 500 μL) methanol solution with agitation. After further agitation for 2 h, DSPE-AH-PEG containing pH-sensitive acylhydrazone bond was added to stabilize the nanoparticles. The excess Mn^2+^ and R848 were removed by ultrafiltration (3 kDa, 5000 rpm) to obtain the PRM nanoparticles. To prepare PARM, Raw 264.7 cells were pre-exposed to ultraviolet radiation to induce the production of apoptotic vesicle. The apoptotic vesicle obtained were freeze–thawed to prepare apoptotic vesicle membrane, mixed with DSPE-AH-PEG, and then co-extruded through 200 and 100 nm filter membrane with RM to obtain PARM^42^^,^^43^.

The particle size and zeta potential of PRM and PARM were determined by Malvern particle size analyzer. The morphology of PRM and PARM was observed by transmission electron microscope (TEM). SDS-PAGE was used to assess the successful modification of the apoptotic vesicle membrane on the nanoparticles. Samples of PRM, PARM and apoptotic vesicle membrane were prepared, followed by the addition of loading buffer to the samples. Subsequently, sampling, electrophoresis, staining, repeated elution, and exposure imaging were performed. To verify the pH-response character of PARM, the pH of PARM solution was adjusted to 6.5. The particle size was measured, and the morphology was observed by TEM. In addition, PARM in pH = 7.4 and 6.5 solutions were incubated with FITC-Annexin V for 30 min, and the flow cytometry was used to detect the FITC-Annexin V binding rate.

### 2.7 Cell uptake

To trace nanoparticles, the fluorescent dye ICG was added to PRM or PARM nanoparticles to synthesize fluorescently labeled nanoparticles, which were respectively named PIRM and PAIRM. Raw 264.7 cells (1 × 10^5^/well) were transferred to a 12-well plate, then PIRM, PIRM (pH = 6.5), PAIRM, and PAIRM (pH = 6.5) were added respectively (*C*_ICG_ = 10 μg/mL), and incubated together for 4 h. The old medium was discarded, cells were collected, and the uptake of nanoparticles by cells was analyzed by flow cytometry. Raw 264.7 cells (5 × 10^4^/well) were inoculated in a 35 mm confocal dish and incubated with different nanomaterials (*C*_ICG_ = 10 μg/mL) for 4 h. Following washing, fixation, and DAPI staining of nuclei, the cells were imaged by CLSM.

To explore the difference of PARM uptake between macrophages and tumor cells, Raw 264.7 cells (5 × 10^4^/well) and 4T1 cells (5 × 10^4^/well) were incubated with PAIRM (pH = 6.5) for 4 h (C_ICG_ = 10 μg/mL), respectively. Then the cells were fixed with 4% paraformaldehyde, stained with DAPI, and observed with CLSM. In the cell co-culture experiment, equal amounts of Raw 264.7 cells (2 × 10^4^) and 4T1-GFP cells (2 × 10^4^) were co-cultured in 35 mm confocal dish, and then PAIRM (pH = 6.5) was added (*C*_ICG_ = 10 μg/mL). After incubation for 4 h, Raw 264.7 cells were stained with PE-labeled F4/80 antibody and finally observed with CLSM.

### 2.8 Macrophage repolarization

Raw 264.7 cells (1 × 10^5^/well) were inoculated into 12-well plates with 50 ng/mL IL-4 to induce differentiation for 48 h. A fresh blank medium was replaced and incubated with R848 (1 μg/mL), MnCl_2_ (Mn^2+^, 2 μg/mL), PRM (pH = 6.5), PARM (pH = 6.5) for 12 h. Cells were collected and stained with anti-CD86-APC and anti-CD206-FITC. Flow cytometry was used to detect the polarization of macrophages. Raw 264.7 cells (5 × 10^4^/well) were inoculated in a 35 mm confocal dish and incubated with R848, MnCl_2_, PRM (pH = 6.5), PARM (pH = 6.5) for 12 h. Then the cells were fixed, stained with APC-anti-CD86 and FITC-anti-CD206, and observed using CLSM. In addition, different nanomaterials were added into the M2 phenotype macrophages and incubated for another 12 h. The supernatant of cell medium was collected for the detection of TNF-*α*, IL-6 and NO, respectively.

### 2.9 Cytotoxicity assay

Raw 264.7 cells (1 × 10^4^/well) were transferred to 96-well plates for overnight culture, and serum-free medium containing different concentrations of PARM was added after the cells were attached to the wall for 12 h. Finally, the survival rate of different groups was measured by CCK-8 assay.

### 2.10 Activation of cGAS–STING pathway in macrophages

Raw 264.7 cells (1 × 10^5^/well) were inoculated into 12-well plates, then R848 (1 μg/mL), MnCl_2_ (Mn^2+^, 2 μg/mL), PRM (pH = 6.5), PARM (pH = 6.5) were added into cells and incubated for 12 h. The cells were collected and lysed to obtain total protein, then Western blot assay was used to detected the expression level of different proteins (TBK-1, IRF-3, STING, phosphorylated TBK-1, phosphorylated IRF-3 and phosphorylated STING).

### 2.11 The phagocytosis of tumor cells by macrophages

Raw 264.7 cells (1 × 10^5^/well) were inoculated into 12-well plates, then R848 (1 μg/mL), MnCl_2_ (Mn^2+^, 2 μg/mL), PRM (pH = 6.5), PARM (pH = 6.5) were added into cells and incubated for 12 h. The cells were collected and the protein and mRNA expression levels of SIRP*α* were determined by Western blot assay and qPCR, respectively. To evaluate the phagocytic ability of macrophages, the Raw 264.7 cells (7.5 × 10^4^/well) were inoculated into 12-well plates, then R848 (1 μg/mL), MnCl_2_ (Mn^2+^, 2 μg/mL), PRM (pH = 6.5), PARM (pH = 6.5) were added into cells and incubated for 12 h 4T1-GFP cells (2.5 × 10^4^/well) were then co-cultured with these Raw 264.7 cells for 4 h (4T1-GFP cells: Raw 264.7 cells = 1:3). The mixture of cells was collected and macrophages were stained with F4/80-PE for flow cytometry assay. The PE and FITC double-positive cell populations were macrophages that phagocytized tumor cells, and the phagocytosis rate was calculated as Eq. (1):

(1) Phagocytosis rate (%) = *Q*2/(*Q*1 + *Q*2) × 100

where *Q*1 and *Q*2 are the corresponding cell proportion values in the flow scatter plot. Meanwhile, the phagocytic ability of macrophage was observed by CLSM.

### 2.12 Antitumor efficacy of PARM *in vivo*

The tumor-bearing mouse model was obtained by subcutaneous injection of 2 × 10^6^ 4T1 cells into the right flank of female BALB/c mice. The mice were intravenously injected with PIRM and PAIRM when tumor volume reached 100–200 mm^3^. Fluorescent images at different timepoint after injection were captured by IVIS Spectrum. Additionally, major organs from both groups of mice were harvested 24 h post-injection of different materials for *ex vivo* fluorescence imaging.

For the *in vivo* therapy, 4T1 tumor-bearing mice were divided into 5 groups randomly with 5 mice in each group. The specific groups were as follows: (I) PBS; (II) R848 (1 mg/kg); (III) MnCl_2_ (Mn^2+^, 2 mg/kg); (IV) PRM; (V) PARM. Beginning on Day 7, treatments were administered every 3 days for a total of 3 doses. Tumor burden (volume and body weight) was monitored and recorded every 2 days. On Day 21, the tumor tissues were obtained from sacrificed mice for histological and immunohistochemical analysis to evaluate therapeutic efficacy.

### 2.13 *In vivo* immune activation

After three rounds of treatment, mice tumor tissues were harvested. Tumor tissues were digested and tissue digests were filtered with a 70 μm filter and washed. Cell suspensions of the different groups were detected by flow cytometry after staining with different antibodies. Tumor tissues were also used for immunofluorescence staining to detect macrophage polarization and T cell infiltration. In addition, the supernatant of the tumor tissue digests was obtained for the detection of TNF-*α*, IL-6 and IFN-*γ* by ELISA kit, respectively. The lung metastasis model of breast cancer was constructed to further verify the potential anti-metastasis effect of PARM. The 4T1 subcutaneous tumor model was constructed and administered three times as described above. Then 5 × 10^5^ 4T1 cells were injected into mice through the tail vein on Day 14. Finally, on Day 28, lung tissues were harvested from the lung metastasis model mice for quantification of lung metastatic nodules and H&E staining.

### 2.14 *In vivo* biosafety and biocompatibility evaluation

After treatments, the main organs and blood of mice from different groups were collected to evaluate the biosafety and biocompatibility of PARM *in vivo*. The main organs were evaluated using H&E staining. Serum was obtained from the collected blood samples by centrifugation. And the levels of aspartate aminotransferase (AST), alanine aminotransferase (ALT), *γ*-glutamyl transpeptidase (*γ*-GT), blood urea nitrogen (BUN) and creatinine (CREA) in different treatment groups were further measured.

### 2.15 Statistical analysis

A minimum of three biological replicates were collected for each experimental group. Data are expressed as mean ± standard deviation (SD). Statistical significance was evaluated by Student's *t*-test and one-way ANOVA, and *P* < 0.05 was considered statistically significant.

## 3 Results and discussion

### 3.1 Downregulation of SIRP*α* by Mn^2+^-mediated cGAS–STING activation

**Figure 2:**
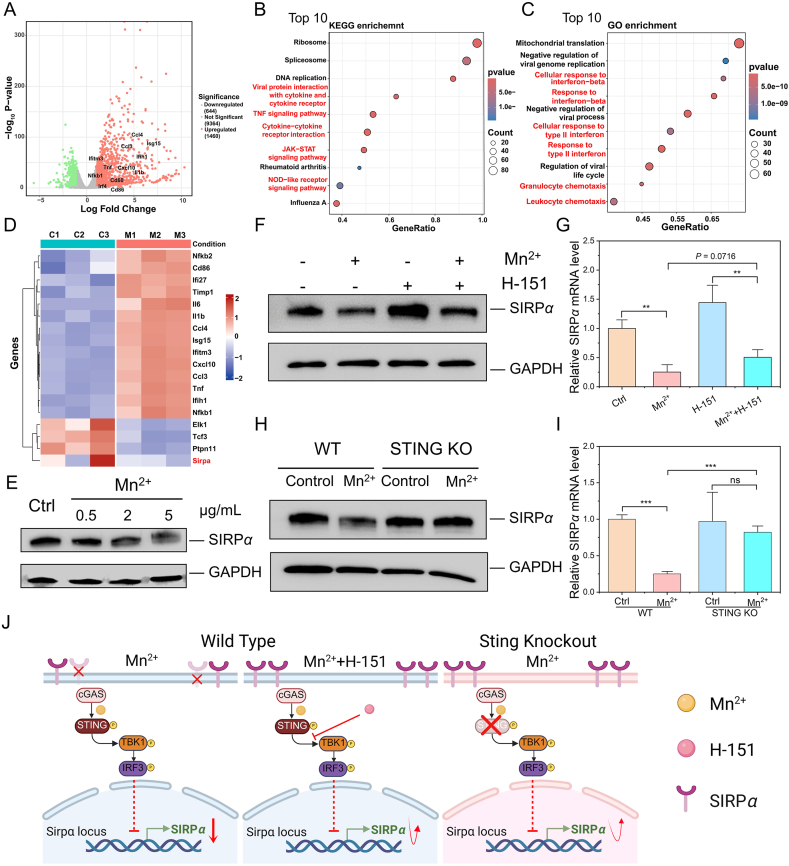
Activation of cGAS–STING pathway by Mn^2+^ down-regulates SIRP*α* expression. (A) Volcano plot of differentially regulated genes in Raw 264.7 cells after Mn^2+^ treatments. (B) KEGG pathway enrichment analysis and (C) GO enrichment analysis of Raw 264.7 cells with Mn^2+^ treatment. (D) Heat map analysis of macrophage proinflammatory polarization, STING activation, and SIRP*α* regulation. (E) Representative expression levels of SIRP*α* protein in Raw 264.7 cells treated with different concentrations of Mn^2+^. (F) Representative SIRP*α* protein expression level and (G) relative SIRP*α* mRNA level in Raw 264.7 cells treated with Mn^2+^ and H-151 (*n* = 3). (H) Representative SIRP*α* protein expression level and (I) relative SIRP*α* mRNA level in WT Raw 264.7 cells and STING KO Raw 264.7 cells treated with Mn^2+^ (*n* = 3). (J) The potential mechanism by which Mn^2+^ activates the cGAS–STING pathway to downregulate SIRP*α* expression, whereas inhibition of cGAS–STING signaling rescues this downregulation. Created in BioRender. Gong, P. (2025) https://BioRender.com/txdd5wr. Data are expressed as mean ± SD. ∗∗*P* < 0.01, ∗∗∗*P* < 0.001; ns, no significance.

Mn^2+^, with its wide availability and low cost coupled with the capacity to strongly activate the cGAS*–*STING pathway, has received extensive attention and been widely used in tumor immunotherapy^44^, ^45^, ^46^. However, it remains unknown whether it can also regulate SIRP*α* by activating the cGAS*–*STING pathway. In this study, the impact of cGAS*–*STING pathway activation by Mn^2+^ on SIRP*α* expression in macrophages was investigated. The transcriptome analysis *via* RNA-seq was performed using Raw 264.7 cells treated with PBS or Mn^2+^. As shown in Fig. 2A, compared with the control group, 1460 genes were significantly up-regulated and 644 genes were significantly down-regulated in Mn^2+^ treatment group. The Kyoto Encyclopedia of Genes and Genomes (KEGG) pathway enrichment analysis and Gene Ontology (GO) enrichment analysis indicated that upregulated genes were mainly related to proinflammatory polarization and cGAS*–*STING activation (Fig. 2B and C). The heat map showed that Mn^2+^ treatment significantly upregulated pro-inflammatory and STING activation related genes, and down-regulated the expression of SIRP*α* gene and its upstream transcription factors *Elk1* and *Tcf3* genes (Fig. 2D).

Western blot analysis was then used to verify Mn^2+^ treatment down-regulation of SIRP*α* expression. As showed in Fig. 2E, the expression of SIRP*α* was significantly decreased following Mn^2+^ treatment. To further investigate the role of cGAS*–*STING pathway, we treated cells with the STING pathway inhibitor H-151, a potent and selective small-molecule inhibitor of STING that covalently binds to a specific cysteine residue within the transmembrane domain of STING, thereby preventing its activation and downstream signaling^47^. Western blot analysis showed that the expression of SIRP*α* in macrophages treated with H-151 + Mn^2+^ was higher than that of the Mn^2+^ treatment group (Fig. 2F). Consistent with the Western blot analysis, the results of quantitative real-time polymerase chain reaction (qPCR) revealed that H-151 treatment attenuated the Mn^2+^-induced reduction in SIRPα expression (Fig. 2G). These findings demonstrate that cGAS*–*STING pathway activation can indeed down-regulate the expression of SIRPα, whereas SIRP*α* expression can be restored *via* targeted inhibition of cGAS*–*STING pathway. To further verify this suggested mechanism, we constructed a STING knockout (KO) cell line by knocking out the STING gene in Raw 264.7 cells, and verified the KO effect of STING by Western blot analysis (Supporting Information Fig. S1). Compared with wild-type (WT) Raw 264.7 cells, Mn^2+^ treatment did not reduce SIRPα protein or gene expression in STING KO cell line (Fig. 2H and I). Together, these results indicate that activation of the cGAS–STING pathway down-regulates SIRP*α* expression in macrophages. We then further investigated whether downregulation of SIRP*α* expression is associated with type-I interferon (IFN), a signature downstream product activated by the cGAS*–*STING pathway^48^, ^49^, ^50^. Based on the results of Western blot analysis, neither IFN-*α* nor IFN-*β* can down-regulate the expression of SIRP*α*, suggesting that the expression of SIRP*α* is regulated by the activation of cGAS*–*STING pathway but does not depend on its downstream cytokine type-I IFN signaling (Supporting Information Fig. S2). Collectively, these findings indicate that cGAS*–*STING pathway activation by Mn^2+^ downregulates SIRP*α* expression, while pharmacological or genetic inhibition of this pathway ameliorates SIRP*α* suppression (Fig. 2J)^51^. Compared to expensive, complex, and extracellularly acting CD47/SIRP*α* antibodies, this approach of regulating SIRP*α* expression using low-cost Mn^2+^ represents a more efficient and promising strategy.

### 3.2 Preparation and characterization of PARM

**Figure 3:**
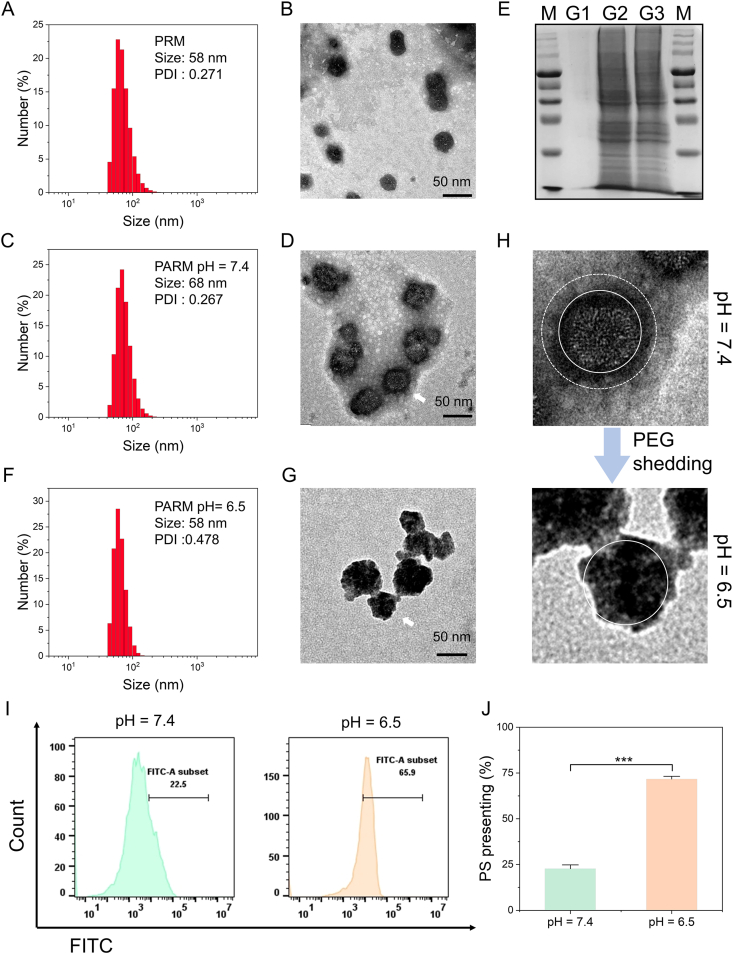
The preparation and characterization of PARM. The particle size of (A) PRM and (C) PARM. The TEM images of (B) PRM and (D) PARM. (E) SDS-PAGE protein analysis of PRM (G1), apoptotic vesicle membrane (G2) and PARM (G3) by Coomassie blue staining. M: marker. (F) The particle size of PARM in a solution at pH = 6.5. (G) The TEM image of PARM in a solution at pH = 6.5. (H) The morphological changes of PARM at pH = 6.5. (I) Flow cytometry analysis and (J) quantification of PS-presenting with FITC–Annexin V staining (*n* = 3). Data are expressed as mean ± SD. ∗∗∗*P* < 0.001.

RM nanoparticles were first formed by coordination between Mn^2+^ and R848^52^. In order to stabilize the nanoparticles, DSPE-AH-PEG with pH-sensitive acylhydrazone bond was added to prepare PRM. PRM has a particle size of about 58 nm and is irregularly spherical in appearance (Fig. 3A and B). RM was extruded with DSPE-AH-PEG and apoptotic vesicle membrane to obtain PEG-capped apoptotic vesicle biomimetic nanoparticles termed PARM. The particle size of PARM is about 68 nm (Fig. 3C), and a membrane shell layer could be observed by TEM images (Fig. 3D). Notably, following coating with apoptotic vesicle membrane, the zeta potential of PARM decreased dramatically from *–*18.8 mV of PRM to *–*35.2 mV (Supporting Information Fig. S3). The particle size of PARM remained stable in both PBS (pH = 7.4) and 10% FBS (Supporting Information Fig. S4). The modification of apoptotic vesicle membrane on PARM was analyzed by SDS-PAGE. As shown in Fig. 3E, PARM exhibits the same protein bands as apoptotic vesicle membrane, while PRM had no corresponding protein bands. Moreover, the particle size of PARM at pH = 6.5 was about 58 nm, which is slightly smaller than the particle size at a physiological pH of 7.4 (Fig. 3F), probably as a result of PEG corona shedding. The PEG-shedding of PARM at pH = 6.5 can be further demonstrated by TEM images. As shown in Fig. 3G and H, compared with PARM at pH = 7.4, the PEG corona around the nanoparticles of PARM (pH = 6.5) highly disappeared. We next simulated the presentation of phosphatidylserine (PS) on the surface of apoptotic vesicle triggered in a slightly acidic TME. As shown in Fig. 3I and J, at a pH = 6.5 solution, 71.7% of the nanoparticles were bound to fluorescein isothiocyanate (FITC)*–*Annexin V, compared to only 22.7% at pH = 7.4 in the control group, suggesting that the acidic TME can facilitate PEG corona shedding on the nanoparticle surface thereby exposing the PS-rich apoptotic vesicle membrane. This provides a possibility for enhancing the uptake of nanoparticles by TAMs.

### 3.3 Macrophage specific targeting *in vitro*

**Figure 4:**
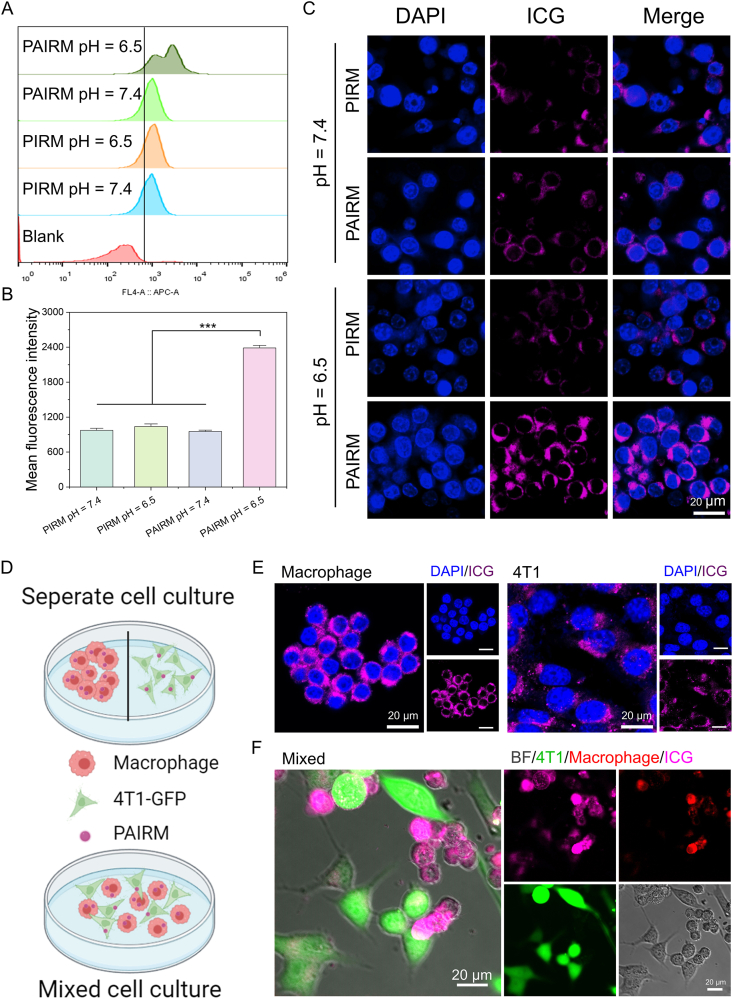
Specific targeting ability to macrophages *in vitro*. (A) Flow cytometry and (B) quantitative analysis of cell uptake after 4 h of co-incubation of different materials (*n* = 3). (C) CLSM images of Raw 264.7 cells with different treatments for 4 h. (D) Schematic of separate cell culture and mixed cell culture system. Created in BioRender. Gong, P. (2025) https://BioRender.com/mns44ev. (E) CLSM images of macrophages or 4T1 tumor cells treated with PAIRM at pH = 6.5 for 4 h. (F) CLSM images of mixed macrophages and 4T1-GFP tumor cells treated with PAIRM at pH = 6.5 for 4 h. Purple: ICG, Red: macrophages. Green: 4T1-GFP tumor cells. Data are expressed as mean ± SD. ∗∗∗*P* < 0.001.

In order to trace the nanoparticles, fluorescent dye indocyanine green (ICG) was added to PRM and PARM nanoparticles to synthesized PIRM and PAIRM, respectively. The ability of PAIRM nanoparticles to target macrophages *in vitro* was evaluated by flow cytometry and confocal laser scan microscopy (CLSM). As shown in Fig. 4A, the cells incubated with PAIRM at pH = 6.5 showed the strongest fluorescence intensity and the ICG intensity in the cells of PAIRM (pH = 6.5) group was about twice that of the other groups (Fig. 4B). As shown in Fig. 4C, the cells incubated with PIRM (pH = 7.4), PIRM (pH = 6.5) and PAIRM (pH = 7.4) showed weak fluorescent signal. In contrast, the cells incubated with PAIRM (pH = 6.5) showed markedly higher ICG fluorescence. These results suggest that the PEG corona of the nanoparticles is lost in the acidic TME thereby targeting TAMs. In contrast, while in the blood circulation or normal tissues, the PEG corona is retained thereby protecting the nanoparticles from uptake by macrophages, improving their circulation time and safety *in vivo*. After the nanoparticles were incubated with macrophages or 4T1 tumor cells for 4 h stronger fluorescence signals were observed in macrophages than in 4T1 tumor cells (Fig. 4E). Moreover, a co-culture system of macrophages and 4T1-GFP tumor cells was established to further assess the targeting ability of PAIRM at pH = 6.5 (Fig. 4D). As shown in Fig. 4F and Supporting Information Fig. S5, an obvious fluorescence signal of ICG was observed in macrophages (red), but not in 4T1tumor cells (green), indicating the feasibility of specific targeting of TAMs in the TME by nanoparticles with apoptotic vesicle membrane encapsulated within the pH-sensitive sheddable PEG corona.

### 3.4 Repolarization of M2-phenotype macrophages *in vitro*

**Figure 5:**
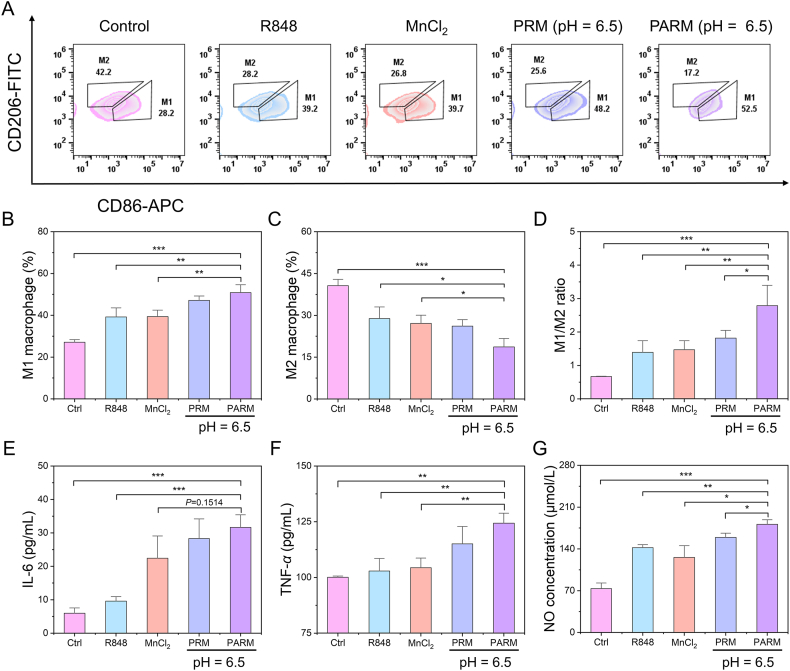
Repolarization of M2-phenotype macrophages *in vitro*. (A) Flow cytometry analysis of CD206 (M2 marker) and CD86 (M1 marker) in macrophages incubated with different materials. The proportion of macrophages with (B) M1 and (C) M2 phenotype (*n* = 3). (D) The M1/M2 ratio of macrophages upon different treatments (*n* = 3). The levels of (E) TNF-*α*, (F) IL-6 and (G) NO secreted by macrophages upon different treatments (*n* = 3). Data are expressed as mean ± SD. ∗*P* < 0.05, ∗∗*P* < 0.01, ∗∗∗*P* < 0.001.

Based on the ability to simultaneously deliver TLR 7/8 agonist R848 and cGAS*–*STING pathway agonist Mn^2+^ to TAMs, PARM has the potential to enable efficient macrophage reprogramming to enhance cancer immunotherapy. Flow cytometry analysis was performed to detect the expression of M1-and M2-related markers. As depicted in Fig. 5A, Raw 264.7 cells incubated with PARM (pH = 6.5) showed an elevated expression of CD86 and decreased expression of CD206. Consequently, the proportion of M1 macrophages increased to 50.8%, while M2 macrophages decreased to 18.7%, significantly lower than other groups (Fig. 5B and C). Macrophages treated with PARM (pH = 6.5) displayed the highest M1/M2 ratio, attaining a ratio of 2.8 (Fig. 5D). CLSM images further confirmed that cells treated with PARM at pH = 6.5 displayed greater M2 to M1 macrophage polarization (Supporting Information Fig. S6). PARM (pH = 6.5) treatment significantly enhanced the secretion levels of tumor necrosis factor-*α* (TNF-*α*), interleukin-6 (IL-6) and nitric oxide (NO), all of which are known to be produced and secreted by M1 macrophages (Fig. 5E*–*G). These findings strongly suggest that PARM effectively induces a phenotypic switch from M2 to M1 macrophages. Additionally, CCK-8 assays demonstrated that PARM exhibits negligible cytotoxicity towards Raw 264.7 cells (Supporting Information Fig. S7), indicating that PARM can promote macrophage polarization towards an anti-tumor phenotype without compromising the viability of immune cells.

### 3.5 Enhanced phagocytosis of macrophage *in vitro*

**Figure 6:**
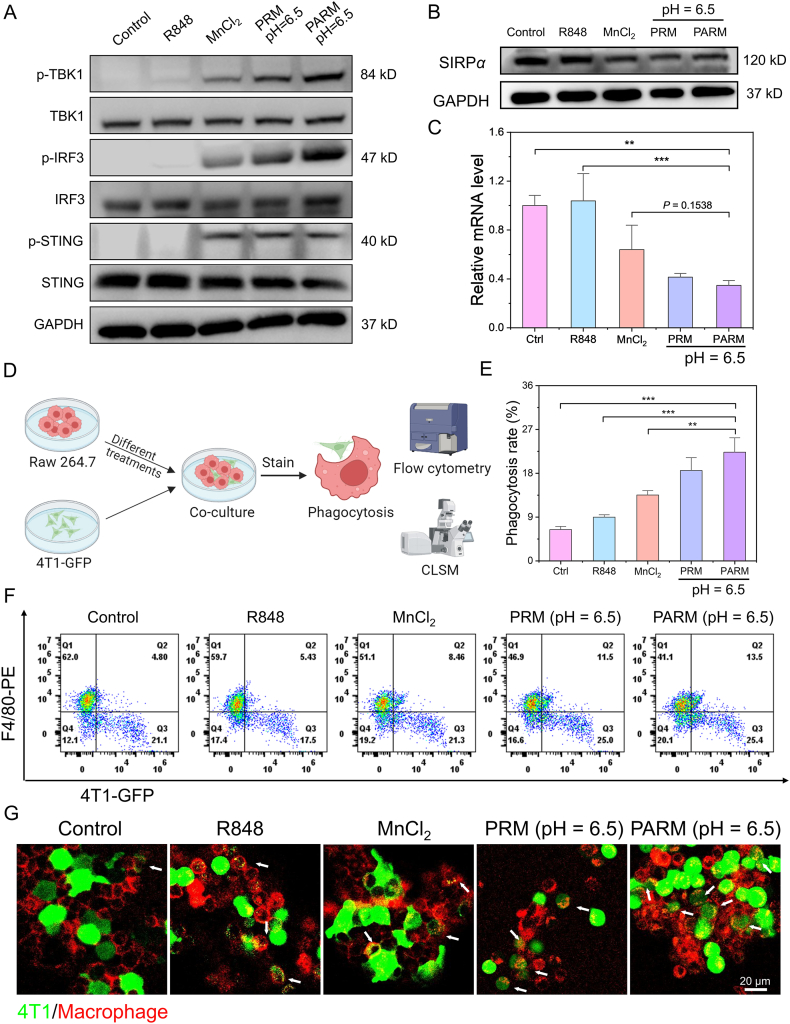
Enhanced phagocytosis of tumor cells by macrophages *in vitro*. (A) Western blot analysis of the expression levels of proteins associated with the activation of the cGAS–STING pathway. (B) The levels of SIRP*α* protein in macrophages under different treatments. (C) Relative SIRP*α* mRNA level in macrophages co-incubated with different materials (*n* = 3). (D) Schematic diagram illustrating the detection of phagocytosis of 4T1 tumor cells by macrophages using flow cytometry and CLSM. Created in BioRender. Gong, P. (2025) https://BioRender.com/mns44ev. (E) Quantitative and (F) flow cytometry analysis of phagocytosis of 4T1 tumor cells by macrophages (*n* = 3). (G) CLSM images of phagocytosis of 4T1-GFP cells (green) by PE-F4/80 antibody-labeled macrophages (red) after different treatments. The white arrow indicates macrophages that phagocytic 4T1 tumor cells. Data are expressed as mean ± SD. ∗∗*P* < 0.01, ∗∗∗*P* < 0.001.

Mn^2+^ has been previously shown to downregulate SIRP*α* expression through activation of cGAS*–*STING pathway. Next, we explored the effects of nanoparticle PARM on activation of the cGAS*–*STING pathway and regulation of SIRP*α*. As shown in Fig. 6A, free Mn^2+^, PRM and PARM activated the cGAS*–*STING pathway. Phosphorylated TBK1, IRF3 and STING protein levels increased significantly in these treatment groups (Supporting Information Fig. S8). In addition, the content of IFN-*β* secreted by macrophages in the PARM (pH = 6.5) treatment group was significantly increased compared with other control groups (Supporting Information Fig. S9). Due to activation of the cGAS-STING pathway, free Mn^2+^, PRM and PARM all induced down regulation of the expression of SIRP*α* protein (Fig. 6B). SIRP*α* mRNA expression was quantified by qPCR, and the results were consistent with those of Western blot analysis (Fig. 6C). Since the CD47/SIRP*α* pathway is closely related to the phagocytosis of macrophages^53^^,^^54^, down-regulation of SIRP*α* expression in macrophages could enhance the phagocytosis of tumor cells by macrophages, hence augmenting the anti-tumor effect. To assess the phagocytic capacity of macrophages, a co-culture system of 4T1-GFP tumor cells and macrophages was established, and flow cytometry and CLSM were used to assess the phagocytic activity of macrophages toward tumor cells (Fig. 6D). The results demonstrated that macrophages treated with free Mn^2+^, PRM, and PARM exhibited enhanced phagocytic activity towards tumor cells. Notably, macrophages treated with PARM at pH = 6.5 displayed the highest phagocytic efficiency, attaining 22.3% (Fig. 6E and F). CLSM further corroborated these findings. As shown in Fig. 6G, macrophages in the PARM (pH = 6.5) treated group showed the strongest phagocytosis, as evidenced by the maximum overlap of red macrophages with green 4T1-GFP tumor cells seen in this group.

### 3.6 Antitumor efficacy of PARM *in vivo*

**Figure 7:**
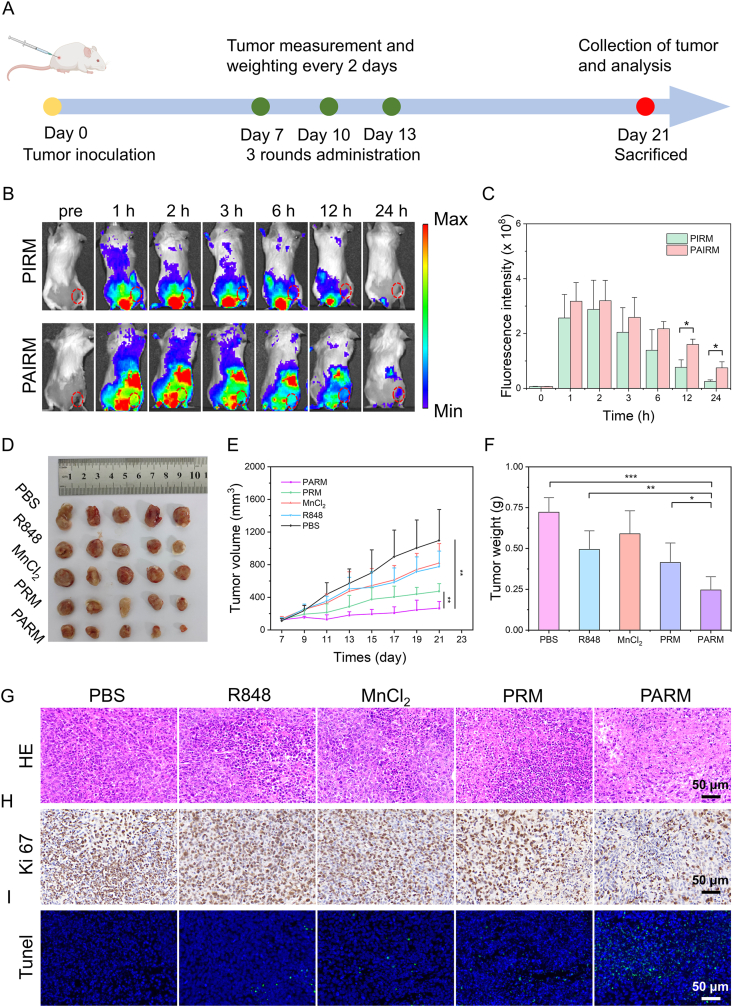
*In vivo* antitumor assessment. (A) The *in vivo* treatment schedule. (B) Fluorescent imaging of mice after injection with PIRM or PAIRM at different time points, tumor sites are indicated by red circles. (C) Semi-quantitative statistical analysis of the tumor sites' fluorescence intensity across different time points. (D) Representative images of tumors collected from mice subjected to different treatments. (E) The tumor volume changes of mice after different treatments in 14 days (*n* = 5). (F) The weight of tumors from mice upon different treatments (*n* = 5). (G) H&E, (H) Ki 67 and (I) Tunel staining of tumor tissues from mice receiving different treatments. Data are expressed as mean ± SD. ∗*P* < 0.05, ∗∗*P* < 0.01, ∗∗∗*P* < 0.001.

Encouraged by the excellent performance of PARM in enhancing macrophage repolarization and phagocytosis *in vitro*, the *in vivo* anti-tumor immunotherapy was assessed according to the treatment regimen shown in Fig. 7A. To explore the biological distribution of PARM, a fluorescence imaging study was carried out. As depicted in Fig. 7B and C, both PIRM and PAIRM could rapidly accumulate in tumor sites within 1 h, and reached the maximum at 2 h after intravenous injection. Fluorescence signal in PAIRM-treated mouse tumor tissue was still detectable 24 h after injection, while there was almost no fluorescence signal in tumor tissue of the PIRM group. For the *ex vivo* biodistribution, the fluorescence signals of both the PIRM- and PAIRM-treated mice groups were mainly concentrated in the kidney and liver, and the fluorescence signal of tumor tissue in PAIRM-treated group was stronger than that of PIRM-treated group (Supporting Information Fig. S10). These observations suggest that the sheddable PEG corona could readily dissociate from the nanoparticles, thereby exposing apoptotic vesicle membrane in acidic TME, leading to enhanced uptake of nanoparticles by TAMs and increased accumulation within the tumor.

In addition, the *in vivo* antitumor efficacy was evaluated by injecting different drugs into the tail vein of tumor-bearing mice. Compared with PBS group, free drug treatment group (R848 and MnCl_2_) achieved a weak therapeutic effect. In contrast, the PRM treatment achieved a good therapeutic effect and inhibited tumor growth. Due to its ability to target reprogrammed TAMs, PARM treatment achieved the best therapeutic efficacy and was the most effective in inhibiting tumor growth (Fig. 7D and Supporting Information Fig. S11). As shown in Fig. 7E and F, the tumor volume and weight were markedly reduced in PARM-treated group compared to other treatment groups, with an average tumor weight of only 0.25 g. Moreover, the PBS, R848, and MnCl_2_ groups exhibited an intense H&E and Ki-67 staining, indicating a high degree of tumor cell proliferation and minimal apoptosis. PRM treatment resulted in decreased tumor cell density and increased apoptosis in tumor tissues. Furthermore, PARM treatment exhibited the most pronounced activity, characterized by significantly reduced tumor cell density and the highest apoptosis ratio, providing compelling evidence for the superior anti-tumor efficacy of PARM *in vivo* (Fig. 7G*–*I and Supporting Information Fig. S12).

### 3.7 Immune activation and lung metastasis inhibition by PARM

**Figure 8:**
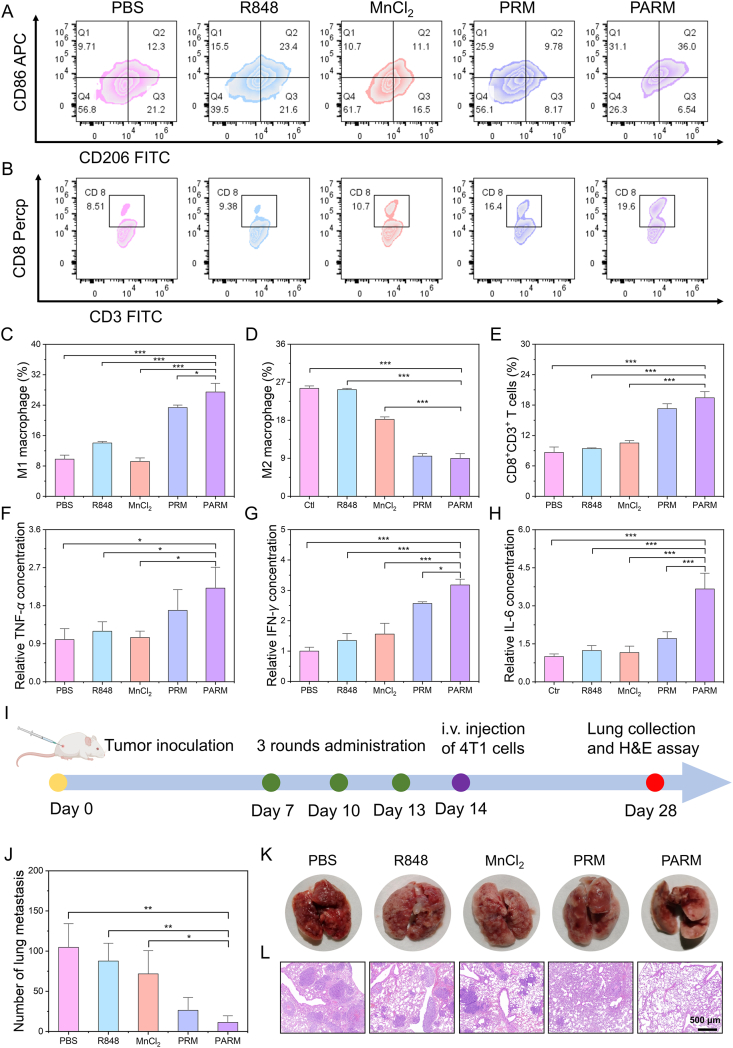
*In vivo* immune activation and inhibition of lung metastasis by PARM. (A) Flow cytometry analysis of the proportions of M1-and M2-polarized macrophages in tumor tissues from mice subjected to different treatments. (B) Flow cytometric analysis of CD8 CD3 double positive T cells in tumor tissues post-treatment. Quantitative analysis of the ratios of (C) M1, (D) M2 macrophages, and (E) CD8^+^CD3^+^ T cells in tumor tissues (*n* = 3). Relative concentration levels of (F) TNF-*α*, (G) IFN-*γ* and (H) IL-6 in tumor tissues from mice subjected to different treatments (*n* = 3). (I) Schematic diagram of the experimental design for the breast cancer lung metastasis model. (J) Quantitative analysis of metastatic nodules in lung tissue of mice receiving different treatments (*n* = 3). (K) Representative images of lung tissue from mice receiving different treatments. (L) H&E staining sections of lung tissue from mice with different treatment. Data are expressed as mean ± SD. ∗*P* < 0.05, ∗∗*P* < 0.01, ∗∗∗*P* < 0.001.

To further confirm that the excellent antitumor activity of PARM was induced by the repolarization of TAMs and the remodeling of the immune microenvironment, the infiltration and distribution of immune cells at the tumor sites were evaluated. Flow cytometric analysis revealed that PARM treatment could elevate the proportion of M1 phenotype TAMs to 28.1%, and reduce the proportion of M2 phenotype TAMs to 8.2% (Fig. 8A–C and D). The immunofluorescence images also showed that PARM induced an increase in the number of M1-like TAMs (red) and a decrease in the number of M2 phenotype TAMs (green) (Supporting Information Fig. S13). The reprogramming of TAMs is conducive to the activation of T cells in regulating the immune microenvironment. As shown in Supporting Information Fig. S14, CD8^+^ T cells were most abundant in the PARM-treated group compared to the other groups. Flow cytometry analysis showed the proportion of CD8^+^ positive T cells in the PARM-treated group attained 20.2% (Fig. 8B and E). In addition, increased release of pro-inflammatory factors such as TNF-*α*, IFN-*γ*, and IL-6 further confirmed the activation of the tumor immune microenvironment (Fig. 8F*–*H). Western blotting analysis of tumors collected from different groups indicated that phosphorylation activation of STING, TBK1 and IRF3 occurred in the PARM group. Moreover, the IFN-*β* content in the tumor tissues of the PARM group was significantly higher than that of the other groups. These results jointly indicate that PARM activated the cGAS-STING pathway in tumor tissues and triggered a strong anti-tumor immunity (Supporting Information Figs. S15 and S16). To assess the ability of PARM to inhibit cancer metastasis, the metastatic breast cancer model was constructed by intravenous injection of 4T1 cells (Fig. 8I). On Day 28, lung tissues were harvested and it was found that PARM treatment could effectively reduce the number of lung metastatic nodules (Fig. 8J). H&E staining further confirmed the superior anti-metastatic efficacy of PARM, as evidenced by the reduced metastatic tumor burden in lung tissues (Fig. 8K and L).

### 3.8 Biosafety and biocompatibility of PARM

The *in vivo* biosafety and biocompatibility of PARM was investigated after anti-tumor therapy. As shown in Supporting Information Fig. S17, histopathological analysis of major organs revealed that no inflammation or abnormalities were found in the major organs in any treatment group. Furthermore, no significant changes in body weight were observed among mice subjected to different therapeutic interventions (Supporting Information Fig. S18). Serum of mice receiving different treatments was collected and biochemistry analysis demonstrated that PARM treatment did not induce any adverse effects on hematological parameters compared to the control groups (Supporting Information Fig. S19). Collectively, these findings suggest that PARM exhibits excellent biosafety and biocompatibility. Due to its excellent biosafety and efficient targeted regulation capabilities, PARM is expected to overcome the challenges of clinical translation and holds broad application prospects.

## 4 Conclusions

In this study, a pH-responsive sheddable PEG-shielded apoptotic vesicle-biomimetic R848/Mn^2+^ coordination nanoparticle termed PARM were constructed. The PEG corona on the surface of PARM could dissociate in response to the slightly acidic TME, thus exposing apoptotic vesicle membrane, which enables PARM to specifically target TAMs. By dual activation of TLR 7/8 and cGAS–STING pathway, M2 phenotype TAMs were readily repolarized into pro-inflammatory M1 phenotype, the proportion of M1 macrophages increased from 27% to 50%. And the activation of cGAS–STING pathway down-regulated SIRP*α*, thereby increasing the phagocytic activity of these macrophages against tumor cells by approximately three times. Consequently, PARM significantly inhibited tumor growth and metastasis by remodeling the immunosuppressed TME. Our work evidently provides novel insights into TAMs-centric immunotherapeutic strategies, establishing a paradigm for precision modulation of macrophage plasticity in cancer treatment.

## Author contributions

Yu Liu: Investigation, Validation, Writing - original draft. Chunbai Xiang: Investigation, Validation, Data curation. Yeneng Dai: Investigation, Validation. Chao Li: Investigation, Validation. Michael N. Okeke: Formal analysis, Software. Ting Jiang: Formal analysis, Validation. Xing Yang: Data curation, Validation. Yehuda G. Assaraf: Writing - review & editing. Kai Miao: Resources. Yue Wang: Investigation, Validation. Zhiwei Zhang: Investigation, Validation. Duo Zhang: Software, Supervision. Yaping Li: Supervision, Writing - review & editing. Ping Gong: Project administration, Resources, Supervision. Qi Zhao: Project administration, Resources, Supervision, Writing - review & editing.

## Conflicts of interest

The authors declare no conflicts of interest.

## Appendix A Supporting information

The following is the Supporting Information to this article.
