# Supplementary material for: Engineered apoptotic vesicle mimetics with tunable “eat-me” signaling precisely regulate tumor-associated macrophages for potentiating cancer immunotherapy
Source: Acta Pharm Sin B. 2025 Nov 29;16(4):2498–512. doi: 10.1016/j.apsb.2025.11.032 (PMC13104663; doi:10.1016/j.apsb.2025.11.032)
Supplement: Multimedia component 1 [file mmc1.pdf]

Supporting Information for

Original article

Engineered apoptotic vesicle mimetics with tunable "Eat-Me" signaling precisely regulate tumor-associated macrophages for potentiating cancer immunotherapy

Yu Liu[a,b,†], Chunbai Xiang[b,†], Yeneng Dai[a], Chao Li[b,c], Michael N. Okeke[b,c], Ting Jiang[b,c], Xing Yang[b], Yehuda G. Assaraf[f], Kai Miao[a], Yue Wang[a,e], Zhiwei Zhang[e], Duo Zhang[e,*], Yaping Li[d,*], Ping Gong[b,c,*], Qi Zhao[a,*]

[a]*MoE Frontiers Science Center for Precision Oncology, Cancer Center, Faculty of Health Sciences, University of Macau, Taipa, Macau 999078, China*

[b]*Key Laboratory of Biomedical Imaging Science and System, Chinese Academy of Sciences, State Key Laboratory of Biomedical Imaging Science and System, Guangdong Key Laboratory of Nanomedicine, CAS–HK Joint Lab for Biomaterials, Shenzhen Institutes of Advanced Technology, Chinese Academy of Sciences, Shenzhen 518055, China*

[c]*University of Chinese Academy of Sciences, Beijing 100049, China*

[d]*State Key Laboratory of Drug Research & Center of Pharmaceutics, Shanghai Institute of Materia Medica, Chinese Academy of Sciences, Shanghai 201203, China*

[e]*Faculty of Pharmaceutical Sciences, Shenzhen University of Advanced Technology, Shenzhen 518107, China*

[f]*The Fred Wyszkowski Cancer Research Laboratory, Faculty of Biology, Technion-Israel Institute of Technology, Haifa 3200003, Israel*

Received 9 July 2025; received in revised form 5 September 2025; accepted 26 September 2025

*Corresponding authors.

E-mail addresses: qizhao@um.edu.mo (Qi Zhao), ping.gong@siat.ac.cn (Ping Gong), ypli@simm.ac.cn (Yaping Li), zhangduo@suat-sz.edu.cn (Duo Zhang).

[†]These authors made equal contributions to this work.

Running title: Engineered apoptotic vesicle mimetics precisely regulate TAMs for cancer immunotherapy

Supporting figures

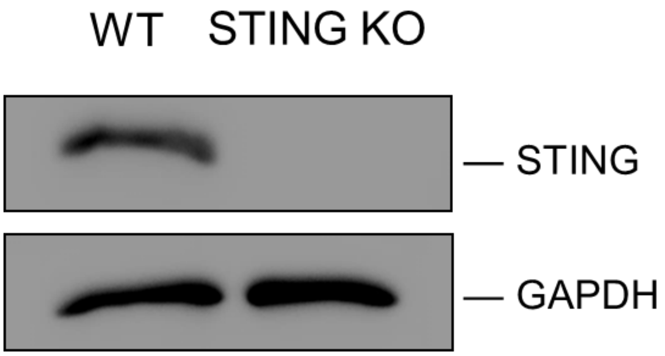

Figure S1 Representative STING protein expression level in WT Raw 264.7 cells and STING KO Raw 264.7 cells.

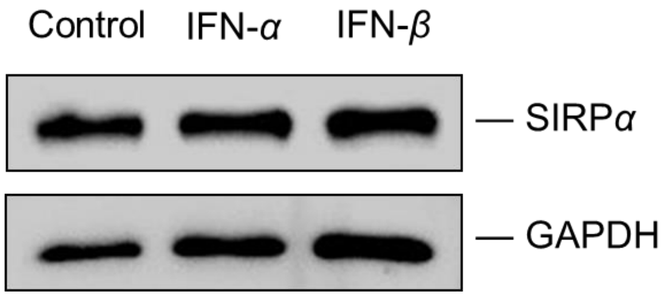

Figure S2 Representative SIRPα protein expression level in Raw 264.7 cells treated with IFN-α and IFN-β.

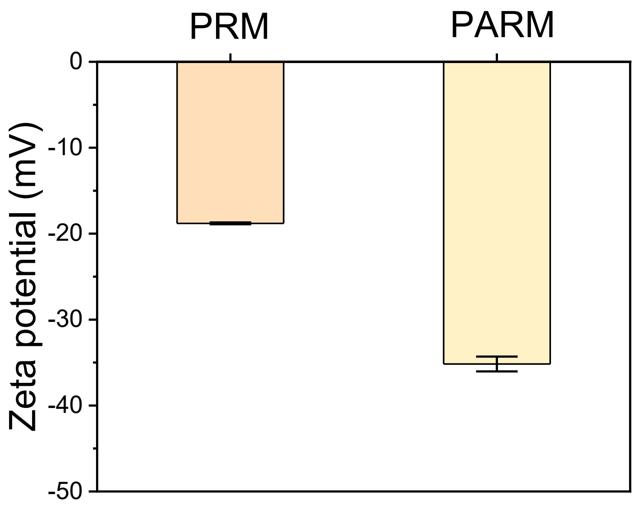

Figure S3 The zeta potentials of PRM and PARM (n = 3). Data are expressed as mean ± SD.

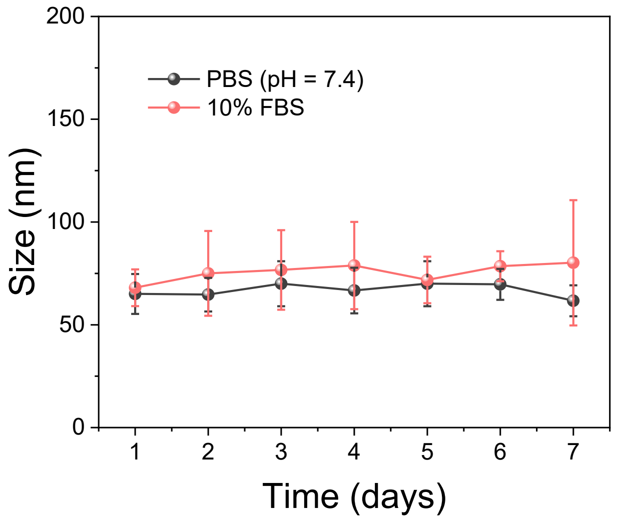

Figure S4 The particle size changes of PARM in PBS (pH = 7.4) and 10% FBS within 7 days (n = 6). Data are expressed as mean ± SD.

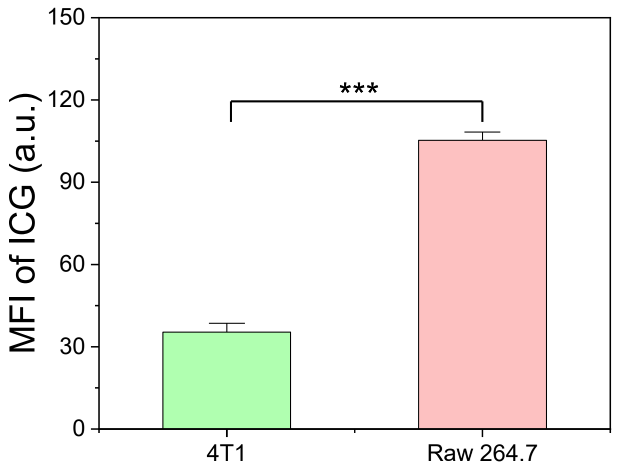

Figure S5 The mean fluorescence intensity (MFI) of ICG in Raw 264.7 cells and 4T1 cells (n = 3). Data are expressed as mean ± SD. ***$P < 0.001$.

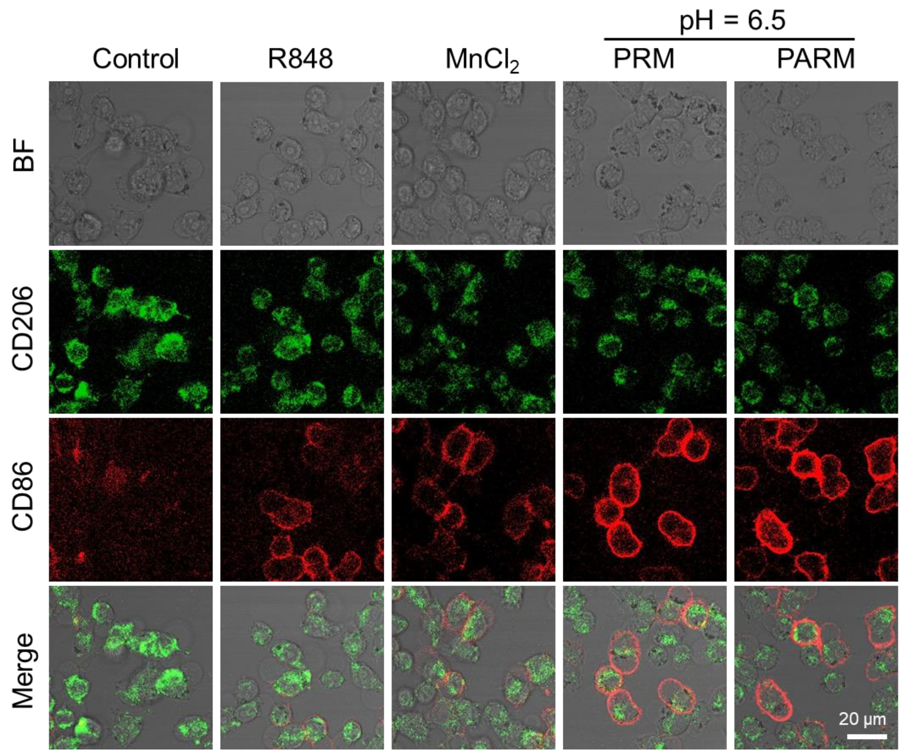

Figure S6 CLSM images of macrophage CD86 and CD206 expression.

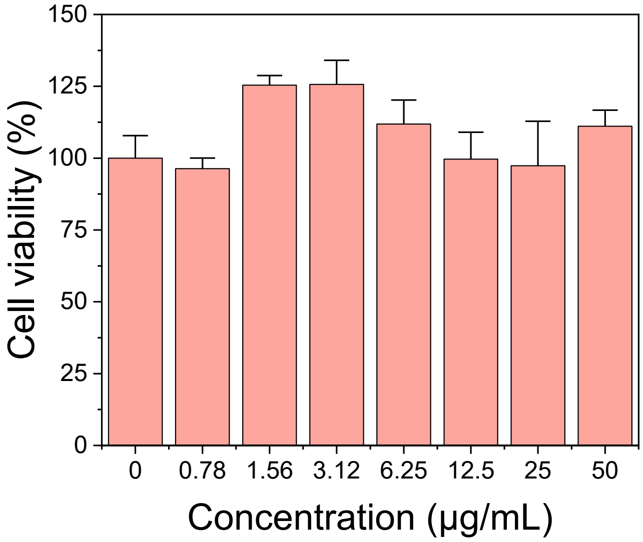

Figure S7 Cell viability of Raw 264.7 cells incubated with PARM at different concentration for 12 h (n = 4). Data are expressed as mean ± SD.

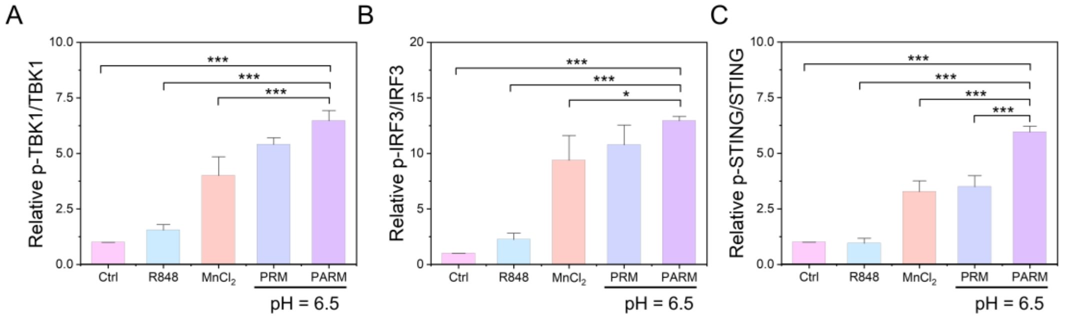

Figure S8 The quantification of phosphorylated protein expression levels of (A) TBK1, (B) IRF3 and (C) STING (*n* = 3). Data are expressed as mean ± SD. $*P < 0.05$, $***P < 0.001$.

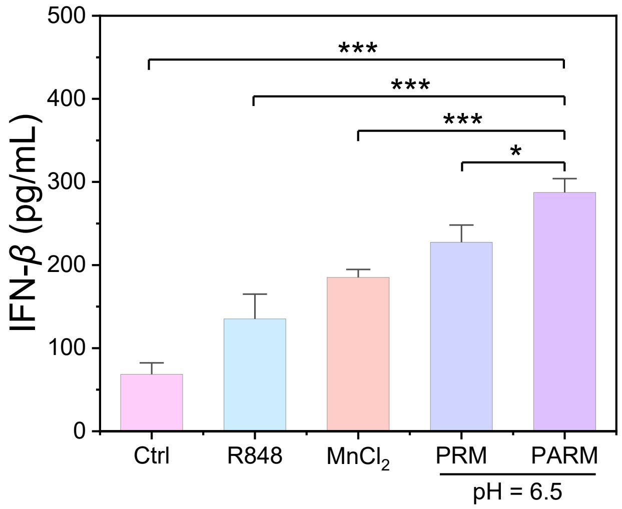

Figure S9 The levels of IFN-*β* secreted by macrophages upon different treatments (*n* = 3). Data are expressed as mean ± SD. $*P < 0.05$, $***P < 0.001$.

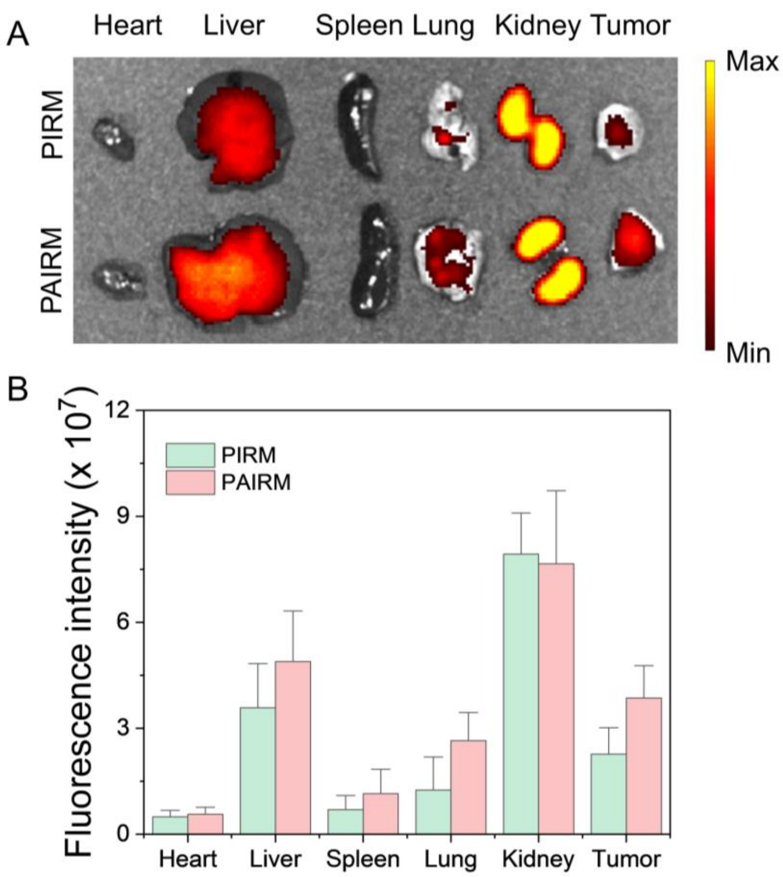

Figure S10 Fluorescence imaging of *ex vivo* major organs. A) Fluorescence images of *ex vivo* major organs of mice 24 h after injection. B) Quantitative fluorescence analysis in major organs from sacrificed mice at 24 h post-injection. Data are expressed as mean ± SD.

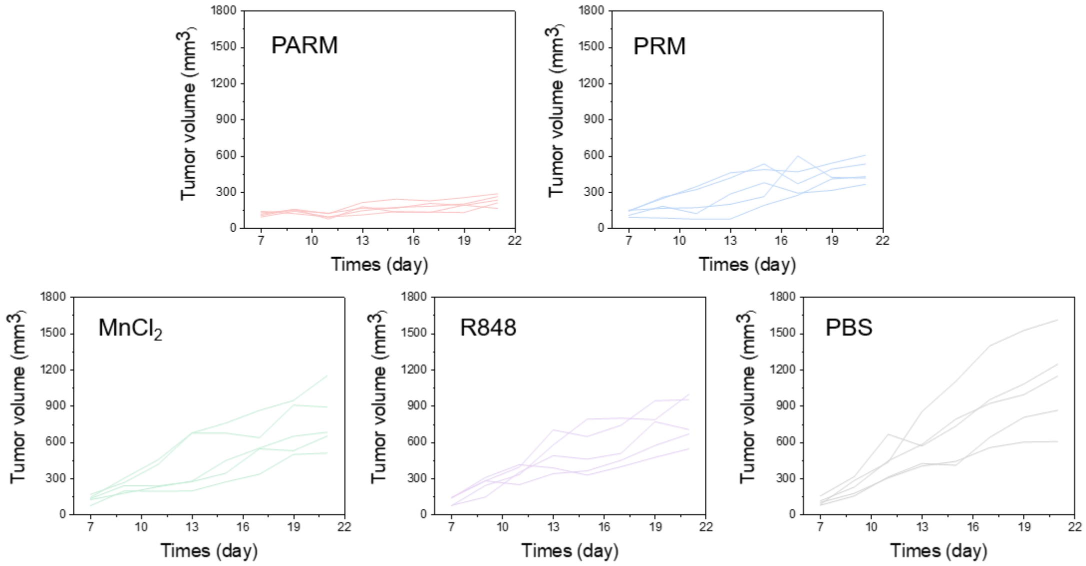

Figure S11 Individual tumor volume growth curves of different groups (n = 5).

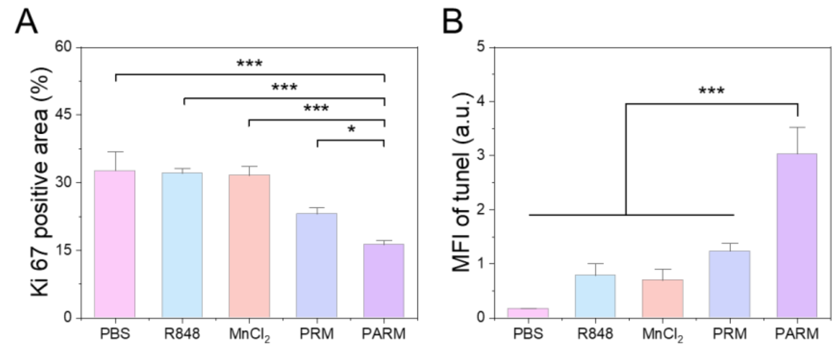

Figure S12 Quantitative analysis of the (A) percentage of Ki67-positive area and (B) mean fluorescence intensity (MFI) of tunel (n = 3). Data are expressed as mean ± SD. *P < 0.05, ***P < 0.001.

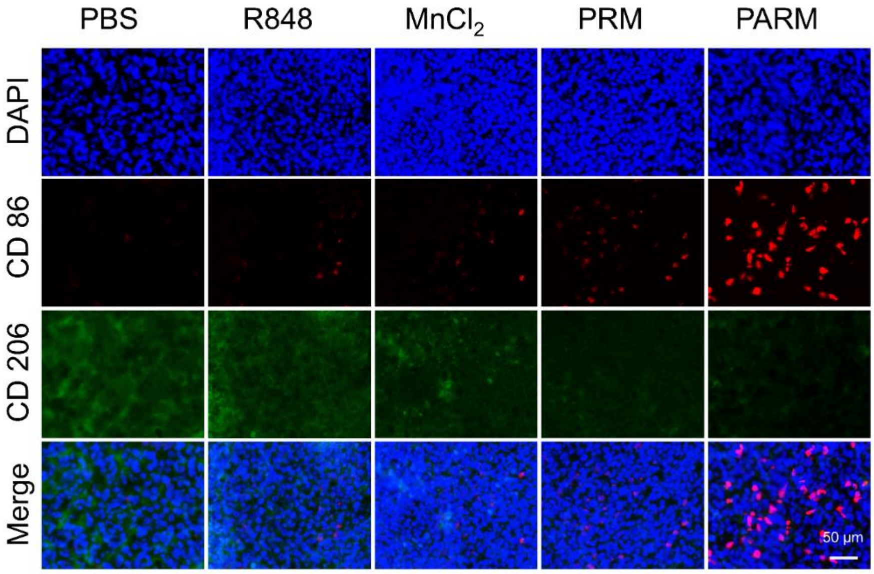

Figure S13 Immunofluorescence analysis of the distribution of macrophages with different phenotypes in tumor tissues.

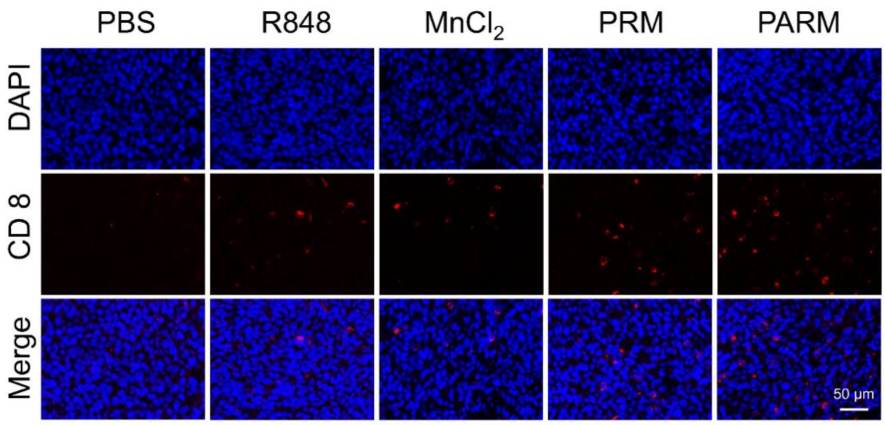

Figure S14 Immunofluorescence analysis of CD8$^+$ T cells distribution in tumor tissues.

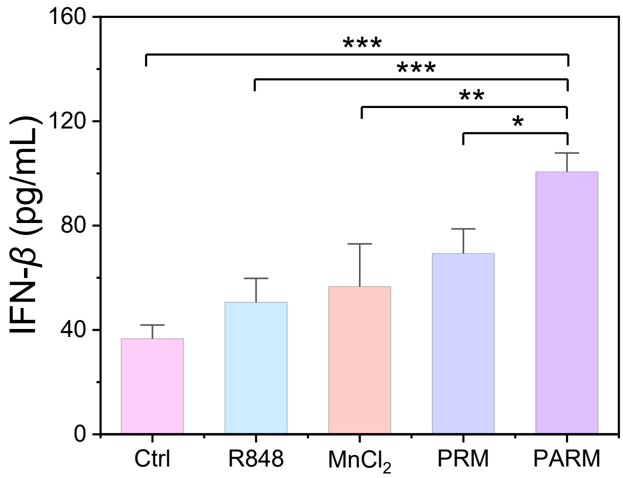

Figure S15 The levels of IFN-β in tumor tissues from mice subjected to different treatments (n = 3). Data are expressed as mean ± SD. *$P < 0.05$, **$P < 0.01$, ***$P < 0.001$.

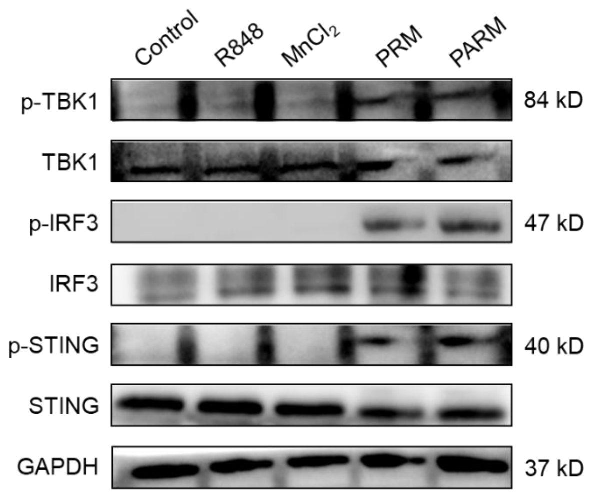

Figure S16 Western blot analysis on the expression of cGAS–STING pathway makers in tumors from 4T1 tumor-bearing mice after different treatments.

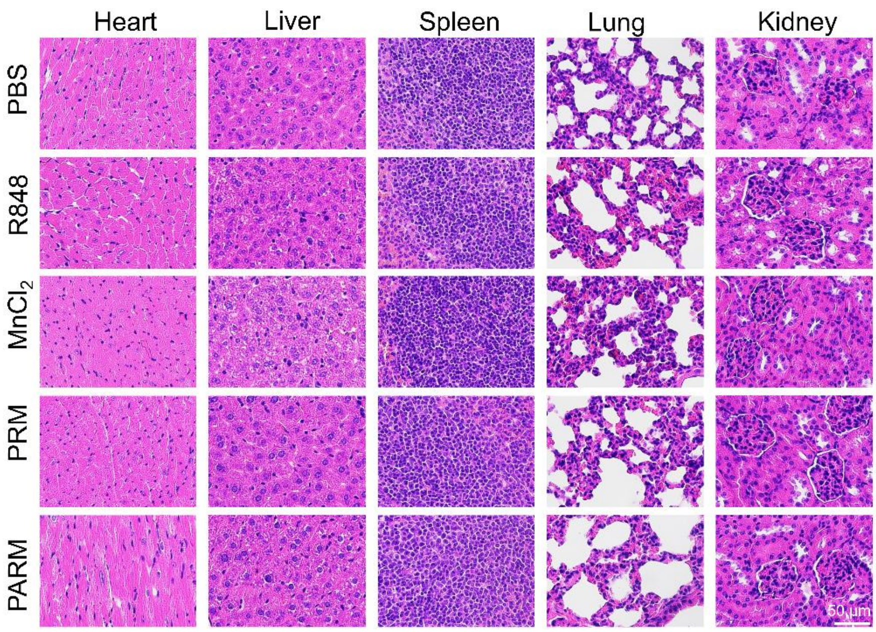

Figure S17 H&E staining of different organs from mice with different treatment groups.

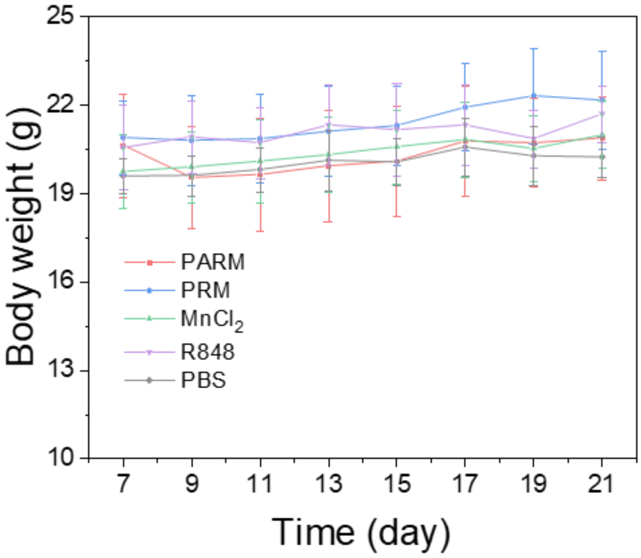

Figure S18 The body weight changes of mice with different treatments (n = 5). Data are expressed as mean ± SD.

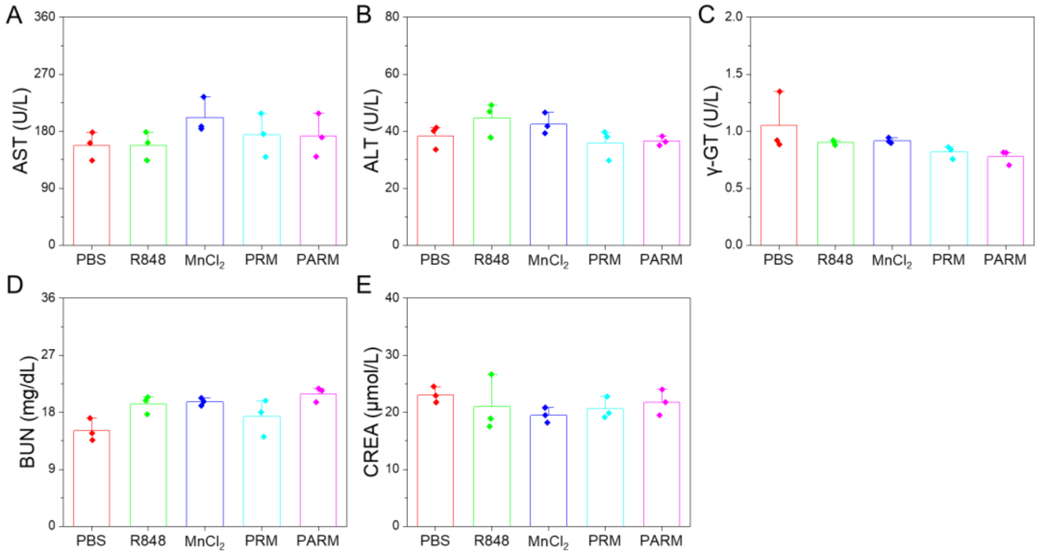

Figure S19 Serum biochemical index levels of mice in different treatment groups. A) Aspartate aminotransferase (AST), B) alanine aminotransferase (ALT), C) γ-glutamyl transpeptidase (γ-GT), D) blood urea nitrogen (BUN) and E) creatinine (CREA) levels in different treatment groups (n = 3). Data are expressed as mean ± SD.
